# Playground slide-related injuries in preschool children: increased risk of lower extremity injuries when riding on laps

**DOI:** 10.1186/s40621-018-0139-x

**Published:** 2018-04-10

**Authors:** Charles A. Jennissen, Maggie Koos, Gerene Denning

**Affiliations:** 1 Department of Emergency Medicine, Roy J. and Lucille A. Carver College of Medicine, University of Iowa, Iowa City, Iowa USA; 2 Children’s Mercy Hospital, Kansas City, MO USA

**Keywords:** Playground slides, Preschool children, Riding on laps, Lower extremity, Fractures, Injury, Public parks

## Abstract

**Background:**

The purpose of this study was to better understand the factors associated with playground slide-related injuries in preschool children and to test the hypothesis that riding on laps increases the likelihood of lower extremity injuries.

**Methods:**

Playground slide-related injuries (product code 1242) in children ≤5 years of age treated in emergency departments from 2002 to 2015 were identified (*N* = 12,686) using the U.S. Consumer Product Safety Commission’s National Electronic Injury Surveillance System (NEISS). Descriptive and comparative analyses, including chi-square testing and binary logistic regression, were performed.

**Results:**

Based on NEISS stratified national sampling estimates, over 350,000 children ≤5 years of age were injured on slides from 2002 to 2015. Overall, 59% of the children were male, and 65% were white. Almost 60% of injuries occurred in parks or other public areas. The most frequent diagnosis was a fracture (36%); lacerations were 19% of the injuries. A higher proportion of musculoskeletal injuries were seen in toddlers < 3 years old as compared to those 3–5 years of age (*p* < 0.001). Injuries to the lower extremities increased in frequency as age decreased, whereas injuries to the upper extremities and head/neck/face were more common in older preschoolers. Children < 3 years of age were 12 times more likely to be identified from narratives as being on another person’s lap at the time of injury. Children identified as being on a lap had an increased odds of injury to the lower extremity than to other body parts (OR 43.0, 95% confidence interval (CI) 32.0–58.0), and of lower leg/ankle fracture than fractures elsewhere (OR 49.5, 95% CI 31.7–77.4).

**Conclusions:**

Decreasing age was associated with a higher likelihood of being identified as sliding down on another person’s lap and a higher likelihood of lower extremity injuries. Healthcare providers should be mindful of the potential for these slide-related injuries as they can result in a toddler’s fracture of the tibia, which may be occult. Parents should also be made aware of this increased risk and counseled that a child’s foot can catch on the slide’s surfaces when going down on a person’s lap with subsequent twisting forces that can result in a fracture.

## Background

Playgrounds play an integral role in the lives of most young children and parents in developed countries. They are a place where children not only exercise and develop their gross motor skills, but also interact with other children to acquire social skills (Frost et al., 2004; Tremblay et al., 2015). In fact, playground equipment has become a cornerstone of elementary schools, daycare centers, public parks, apartment building play areas, and the backyards of many homes. Unfortunately, they also pose a significant risk of injury (Mack et al., 2000; Norton et al., 2004).

Slides are a popular playground and backyard apparatus (Nixon et al., 2003), and have been a major contributor to playground equipment-related injuries (Purvis and Hirsch, 2001; Fuselli and Yanchar, 2012). Over one-fifth of all ED visits for US playground-related injuries are due to slides, and 4.5% of these have required hospitalization (Loder, 2008; Vollman et al., 2009). In Korea, slides accounted for the highest percentage of playground equipment-related injuries resulting in ED visits (40.5%) and hospitalizations (33.9%) (Bae et al., 2017).

Studies focused on playground-related injuries, including those involving slides, have found that the major mechanism of injury was a fall (Vollman et al., 2009; Bae et al., 2017; Bernardo et al., 2001; Mayr et al., 1995; Petridou et al., 2002). These studies varied in terms of the age range of the study population and none looked in detail at other mechanisms. In an editorial, MacKay presented results from a meeting of world experts on playground injuries that included a discussion on the current state of research and existing gaps in knowledge (MacKay, 2003). Attendees concluded that the evaluation of proposed intervention strategies would likely be hampered by knowledge gaps that result in an incomplete understanding of playground-related injuries. These gaps include information on body part injured and age-specific risk factors.

One area for which this knowledge gap exists is in our understanding of slide-related injuries in preschool children. Children < 6 years old may be particularly vulnerable to injury on playgrounds due their poorly developed body control and judgment, and their lack of risk awareness (Schwebel and Brezausek, 2014). One specific mechanism for potential injury in children of this age is traveling down a slide on an older individual’s lap, such as an adult parent. Dr. John Gaffney, a pediatric orthopedic surgeon, studied 58 children with tibia fractures over an 11-month period from 2007 to 2008, and found that eight (14%) had been injured in this manner (Gaffney, 2009). The children ranged in age from 14 to 32 months old.

To our knowledge, this is the only report that has identified this activity, i.e. sitting on laps, as a potential risk factor for slide-related injury. However, a study of 75 toddler’s fractures at Seattle Children’s Hospital from 2008 to 2012 showed that over a third of them were due to the leg being “caught on a slide” (Schuh et al., 2016). Unfortunately, the study did not determine whether the child had been on a lap or not at the time of the injury.

Based on these considerations, the overall objective of this study was to utilize a national database to better understand the factors associated with slide-related injuries in young children. We hypothesized that the slide-related injuries experienced by preschool children 3 years and older, who are for the most part developmentally capable of utilizing playground slides independently, would be quite different from children less than 3 years of age who generally require at least some assistance in utilizing slides. More specifically, we hypothesized that younger children may be more likely to be on an individual’s lap when going down a slide and that being on a lap may increase the likelihood of a lower extremity injury.

## Methods

### National Electronic Injury Surveillance System (NEISS)

This was a retrospective study and data were downloaded from the U.S. Consumer Product Safety Commission (CPSC) NEISS database query page (United States Consumer Product Safety Commission,  n.d.). The CPSC monitors consumer product-related injuries in the US through the NEISS database that compiles information from approximately 100 EDs nationwide. These data represent a stratified probability sampling of ED visits across the country and allows the calculation of national estimates of injuries associated with a given consumer product.

For the database query, the product code 1242 was used to obtain records of children ≤5 years of age that were injured on slides. This code excludes injuries related to swimming pools and water slides. The study covers the time period from 2002 through 2015. The year 2002 was selected for the beginning of the study as that was the year narratives, which often provide a brief description of the activity being performed at the time of the injury, were added to the database. The University of Iowa Institutional Review Board deemed this study exempt.

### Variables coded in NEISS and utilized in the study

Demographic study variables included age, sex, and race. Frequency data are provided for the ages < 1 year old (1-11 months), 1 year old (12–23 months), 2 years old (24-35 months), 3 years old (36–47 months), 4 years old (48–59 months), and 5 years old (60–71 months). Bivariate and regression analysis were performed using two age ranges, < 3 years old (1–35 months) and ≥ 3 years old (36-71 months) selected according to child development criteria.

The time-related study variable used was the year that the injury occurred. The “Location” variable was coded as Private Residence, School/Daycare, and Park/Public Area (Table 1). A search of the narrative using the keyword “Daycare” was performed to verify that cases with this narrative text were coded as School/Daycare; three cases miscoded as “Home” were recoded. Two cases coded as Street/Highway (NEISS code: 4) were not included in location-related analysis. There were no cases coded as Industrial (NEISS code: 7).

**Table 1:** Codes from the NEISS database and how they were grouped for study variables

Variable name	Variable value	NEISS codes
Location	Private Residence	1, 2, 6
	School/Daycare	8
	Park/Public Area	5, 9
Diagnosis	Skin	46–51, 53, 54, 58, 59, 63, 72, 73
	Musculoskeletal	55, 57, 64
	Internal Organ	62
	Brain	52, 56
Body Part	Head/Neck/Face	75–77, 88, 89, 94
	Upper Extremities	30, 32–34, 80, 82, 92
	Lower Extremities	35–37, 81, 83, 93
	Torso	31, 38, 79
Fractures	Lower Leg and Ankle	36, 37
	Other Leg	35, 81, 83, 93
	Upper Extremities	32–34, 80, 82, 92
	Other	30, 31, 75, 76, 79, 89
Disposition	Left ED	1, 6
	Stayed/Transferred	2, 4, 5

NEISS injury-related variables included “Diagnosis”, “Body Part” injured, and “Disposition” from the ED (Table 1). Diagnosis was coded utilizing the NEISS “Diagnosis” codes grouped as the following: Skin (e.g. 59 laceration), Musculoskeletal (e.g. 64 strain/sprain), Internal Organ, and Brain (e.g. 52 concussion). Victims with a NEISS Diagnosis of “Internal Organ” were reclassified as “Brain”, if the body part injured was head (NEISS code 75). Other injuries (e.g. 56 foreign body; NEISS codes: 41, 42, 56, 60, 61, 65, 67, 68, 71, 74) were not included in diagnosis-related analysis.

The body part injured was coded using the NEISS variable “Body Part” and grouped as the following: Head/Neck/Face, Upper Extremities, Lower Extremities, and Torso. Children with fractures were identified through the NEISS “Diagnosis” code 57 and then fracture location was grouped using the variable “Body Part” as follows: Lower Leg and Ankle, Other Leg, Upper Extremities, and Other. The disposition was coded using the NEISS variable “Disposition” as Left ED or Stayed/Transferred. There were no slide-related fatalities in the database.

The NEISS injury narratives were utilized to identify patients who were reported as sitting on the lap of another person at the time of the injury. Two members of the research team independently performed an iterative process coding identified cases and the results were compared to resolve any differences. Keyword searches included all words for a person, e.g. mother, father, cousin, aunt, babysitter, adult, brother, sister, etc. Searches also included the words lap, another, with, caught, and behind. The combined effort identified 644 cases that clearly involved the injured child sitting on another person’s lap while going down the slide.

### Statistical analysis

All variables in the study, including age, were coded as categorical. Descriptive (frequencies, percentages), preliminary bivariate analyses to determine *p* values (Pearson chi square test), and multivariate (binary logistic regression) analyses to determine adjusted odds ratios (aOR) and 95% confidence intervals (95% CI) were performed using SPSS (IBM Statistics Package for the Social Sciences, v22). All *p* values were two-tailed with significance defined as *p* < 0.05. When comparisons of variables with more than two values using SPSS yielded an overall *p* value < 0.05, pairwise (2X2) comparisons to generate unadjusted odds ratios (OR) and 95% confidence intervals (95% CI) were performed using the Vassar website for statistical analysis (vassarstats.net). For regression models, covariates for inclusion in the model were identified from bivariate analyses. Variables that were included in the logistic regression analyses were sex, age, race, location, diagnosis, body part, disposition and whether they were identified as being on a lap at the time of their injury. Adjusted odds ratios (aOR) and 95% CI from regression analysis are presented. National estimates of slide-related injuries were determined by the NEISS as calculated using their sampling weights. Missing data were not included in analyses.

## Results

### Slide-related injuries in preschoolers

The NEISS database query yielded 12,686 cases of slide-related injuries for children ≤5 years old. The overall annual number of slide-related injuries increased slightly over the time course of the study from an annual average of 817 for the years 2002–2008 to an average of 995 for 2009 through 2015. Based on extrapolation of the stratified national sampling of cases in the NEISS database, there were an estimated 352,698 U.S. ED visits by children 5 years of age and younger who were injured on slides from 2002 through 2015.

The majority (59%) of injured children in the database were male and approximately two-thirds were white (Table 2, All injuries). The most common diagnosis was a musculoskeletal injury and the second most common was a skin-related injury. The most frequent specific diagnosis was a fracture (36%), followed by lacerations that comprised 19% of all injuries (data not included in Table 2). The most common body parts injured were the head/neck/face region (37%) and the upper extremities (33%). Just over a quarter of the injuries were to the lower extremity. Over 90% of patients were released from the ED following treatment and no fatalities were recorded. Almost 60% of injuries occurred in a park or public area, approximately one-quarter occurred in a school or daycare setting, and the remainder occurred at a home. A narrative keyword search identified 644 children (5% of cases) who were injured while going down a slide on another person’s lap, usually a parent but also including others such as a grandparent or babysitter.

**Table 2:** Characteristics of all playground slide-related injuries from 2002 to 2015 for children ≤5 years of age in the NEISS database

	All Ages	< 1 years old	1 year old	2 years old	3 years old	4 years old	5 years old
*N* (row %)	12,686	151 (1%)	2746 (22%)	2455 (19%)	2299 (18%)	2415 (19%)	2620 (21%)
Variable	*n* (column %)	*n* (column %)	*n* (column %)	*n* (column %)	*n* (column %)	*n* (column %)	*n* (column %)
Sex							
Male	7448 (59%)	97 (64%)	1612 (59%)	1366 (56%)	1329 (58%)	1460 (61%)	1584 (60%)
Female	5234 (41%)	54 (36%)	1133 (41%)	1088 (44%)	970 (42%)	953 (39%)	1036 (40%)
Race							
White	5998 (65%)	68 (59%)	1422 (74%)	1159 (65%)	1064 (64%)	1091 (62%)	1194 (62%)
Other	3179 (35%)	48 (41%)	511 (26%)	628 (35%)	588 (36%)	665 (38%)	739 (38%)
Diagnosis							
Musculoskeletal	6016 (52%)	97 (74%)	1493 (61%)	1087 (48%)	1021 (48%)	1042 (47%)	1276 (52%)
Brain	1276 (11%)	4 (3%)	232 (10%)	288 (13%)	242 (11%)	240 (11%)	270 (11%)
Skin	4359 (37%)	30 (23%)	708 (29%)	877 (39%)	879 (41%)	948 (43%)	917 (37%)
Internal Organ^a^	9 (0.1%)	0 (0%)	1 (0.04%)	2 (0.1%)	1 (0.1%)	2 (0.1%)	3 (0.1%)
Body Part							
Lower Extremities	3320 (26%)	123 (82%)	1631 (60%)	715 (29%)	349 (15%)	239 (10%)	263 (10%)
Upper Extremities	4166 (33%)	8 (5%)	373 (14%)	681 (28%)	893 (39%)	1015 (42%)	1196 (46%)
Head/Neck/Face	4641 (37%)	14 (9%)	664 (24%)	985 (40%)	953 (42%)	1031 (43%)	994 (38%)
Torso	503 (4%)	5 (3%)	60 (2%)	61 (2%)	90 (4%)	124 (5%)	163 (6%)
Fractures Only^b^							
Lower Leg and Ankle	1354 (30%)	60 (88%)	740 (75%)	319 (40%)	124 (16%)	64 (8%)	47 (4%)
Other Leg	206 (5%)	4 (6%)	50 (5%)	52 (7%)	40 (5%)	25 (3%)	35 (3%)
Upper Extremities	2648 (58%)	3 (4%)	161 (16%)	352 (45%)	522 (68%)	692 (82%)	918 (85%)
Other	319 (7%)	1 (1%)	31 (3%)	65 (8%)	78 (10%)	67 (8%)	77 (7%)
Disposition							
Left ED	12,062 (95%)	149 (99%)	2692 (98%)	2362 (96%)	2174 (95%)	2271 (94%)	2414 (92%)
Stayed/Transferred	622 (5%)	2 (1%)	53 (2%)	93 (4%)	124 (5%)	144 (6%)	206 (8%)
Location							
Private Residence	1636 (18%)	13 (14%)	355 (20%)	353 (21%)	325 (20%)	302 (17%)	288 (15%)
School/Daycare	2094 (23%)	2 (2%)	189 (10%)	274 (16%)	377 (23%)	517 (30%)	735 (37%)
Park/Public Area	5256 (59%)	79 (84%)	1268 (70%)	1076 (63%)	946 (57%)	933 (53%)	954 (48%)
Identified as on Lap^c^							
Yes	644 (5%)	52 (34%)	408 (15%)	113 (5%)	32 (1%)	23 (1%)	16 (1%)
Not Mentioned	12,042 (95%)	99 (66%)	2338 (85%)	2342 (95%)	2267 (99%)	2392 (99%)	2604 (99%)

*Abbreviations*: *ED* emergency department, *NEISS* National Electronic Surveillance System

^a^Not including brain

^b^Children with fractures, *N* = 4527

^c^Coded from narrative and likely underestimates the number of children who were on another person’s lap at the time of the injury

### Age-based comparisons for all injuries

Table 2 also provides frequencies as a function of age and Table 3 shows the results of a bivariate analysis of children < 3 years old versus children 3–5 years of age. A significantly lower proportion of injured children < 3 years of age were male (*p* = 0.014) and a significantly higher proportion were white (*p* < 0.0001) as compared to older children (Table 3). The proportion of injuries that occurred at school and daycare facilities increased with age (Table 2). In this respect, slide-related injuries in children < 3 years of age were about 60% less likely to occur at a school or daycare center and nearly twice as likely to occur at a park or public area as compared to older preschool children (Table 3).

**Table 3:** Comparative analysis of playground slide-related injuries by age group. Younger children (< 3 years old) were compared to children 3–5 years of age using the Pearson chi square test

	Pearson χ^2^ Test		
	OR	95% CI	*p* value
Sex			
Male vs. Female (ref)	0.91	0.85–0.98	0.014
Race			
White vs. Other (ref)	1.33	1.22–1.45	< 0.0001
Diagnosis			
Musculoskeletal vs. Other (ref)	1.31	1.22–1.41	< 0.0001
Brain vs. Other (ref)	0.99	0.88–1.11	0.84
Skin vs. Other (ref)	0.75	0.60–0.81	< 0.0001
Body Part			
Lower Extremities vs. Other (ref)	6.57	6.01–7.19	< 0.0001
Upper Extremities vs. Other (ref)	0.34	0.31–0.37	< 0.0001
Head/Neck/Face vs. Other (ref)	0.66	0.61–0.71	< 0.0001
Torso vs. Other (ref)	0.45	0.36–0.55	< 0.0001
Fractures Only^a^			
Lower Leg and Ankle vs. Other (ref)	16.3	13.8–19.1	< 0.0001
Disposition			
Stayed/Transferred vs. Left ED (ref)	0.41	0.34–0.50	< 0.0001
Location			
Private Residence vs. Other (ref)	1.03	0.92–1.15	0.62
School/Daycare vs. Other (ref)	0.39	0.34–0.44	< 0.0001
Park/Public Area vs. Other (ref)	1.70	1.54–1.87	< 0.0001
Identified as on lap			
Yes vs. No (ref)	12.3	9.56–15.7	< 0.0001

*Abbreviations*: *ED* emergency department, *OR* unadjusted odds ratio, *CI* confidence interval

^a^Children with fractures, *N* = 4527

The proportion of children whose injuries resulted in hospitalization or transfer from the ED significantly increased with age from 1% for children < 1 years old to 8% for 5 year olds (Table 2). Children <3 years of age were about 60% less likely to require hospital admission or transfer as compared to children who were 3–5 years of age (Table 3). As compared to older children in the study, children < 3 years old were more likely to have a musculoskeletal injury and less likely to have skin-related injuries. In addition, children < 3 years of age were less likely to have an injury in the region of the head, neck, and face or the upper extremities, but more than 6.5 times more likely to have injured their lower extremities. The proportion of children with fractures to the lower leg/ankle progressively increased as age decreased (Table 2), and children < 3 years old were 16 times more likely to have a lower leg/ankle fracture than all other fractures as compared to older preschool children (Table 3).

**Fig. 1:**
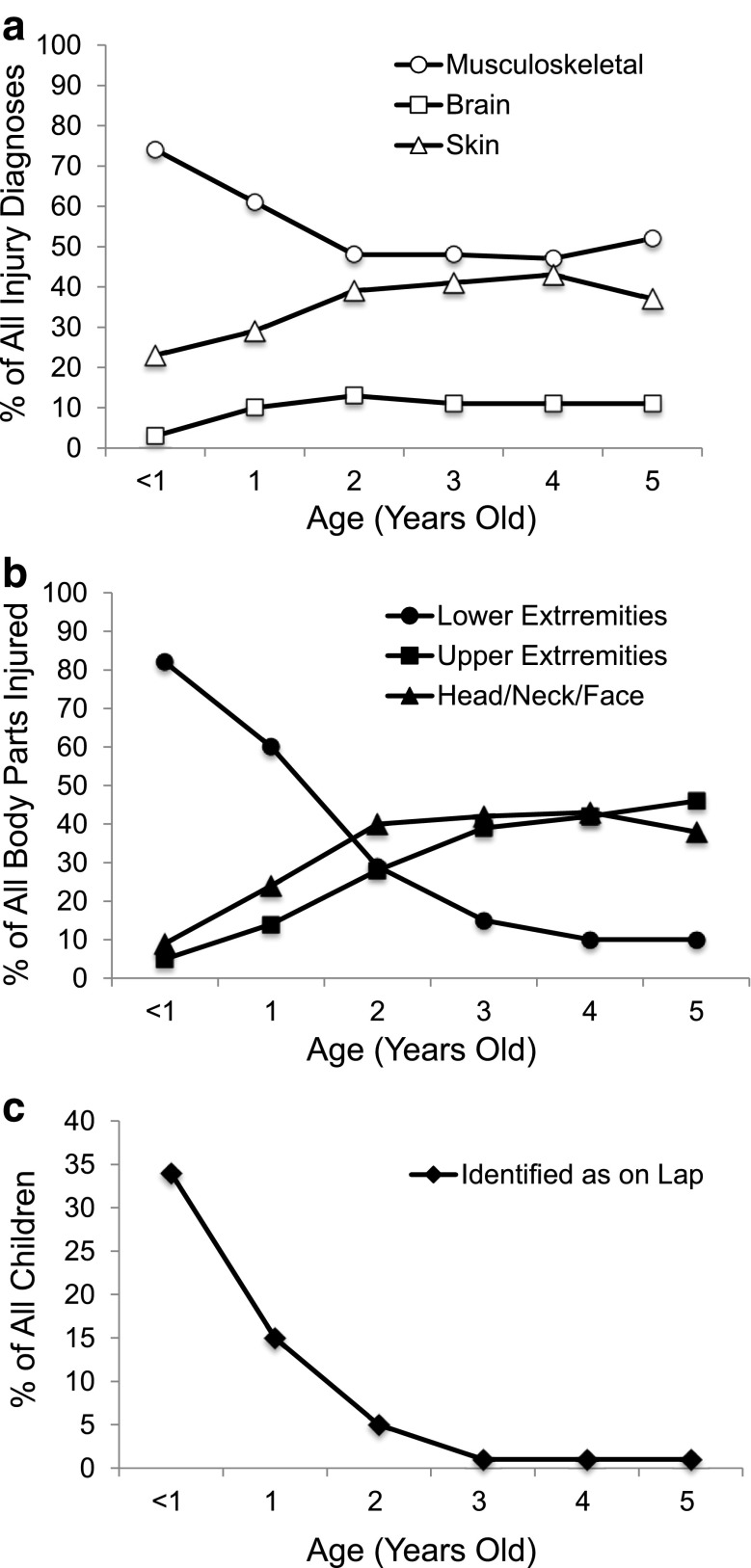
Percent of all injuries as a function of age. The percentage of children from < 1 to 5 years of age with the indicated injury diagnoses (Panel **a**) and the indicated body parts injured (Panel **b**) are shown. Panel (**c**) shows the percent of children of each age that were identified as being on a lap at the time of injury

Almost three-fourths of the injury diagnoses among children < 1 years old were musculoskeletal and the proportion of these injuries decreased with age (Fig. 1a). Comparative analyses showed that the decreases were significant until 2 years of age (all *p* < 0.01). Both the percentage of skin-related (*p* < 0.0001) and brain-related (*p* = 0.0014) injury diagnoses were significantly higher in children 2–5 years of age relative to children < 2 years old. The highest proportion of lower extremity injuries were also observed in children < 1 years old (82%), and this decreased up to 4 years of age (Fig. 1b). Conversely, the percentage of upper extremity injuries increased with every year of age, and the proportion of head/neck/face injuries increased up to 2 years of age.

### Lap-related injuries

As might be expected, younger children were more likely to be identified as sitting on a lap, with proportions decreasing each year until 3 years of age, where only 1% of cases were identified in the narrative as lap-related (Table 2 and Fig. 1c). In all, children < 3 years of age were over 12 times more likely to be identified as being on a lap at the time of injury as compared to older children (Table 3).

**Fig. 2:**
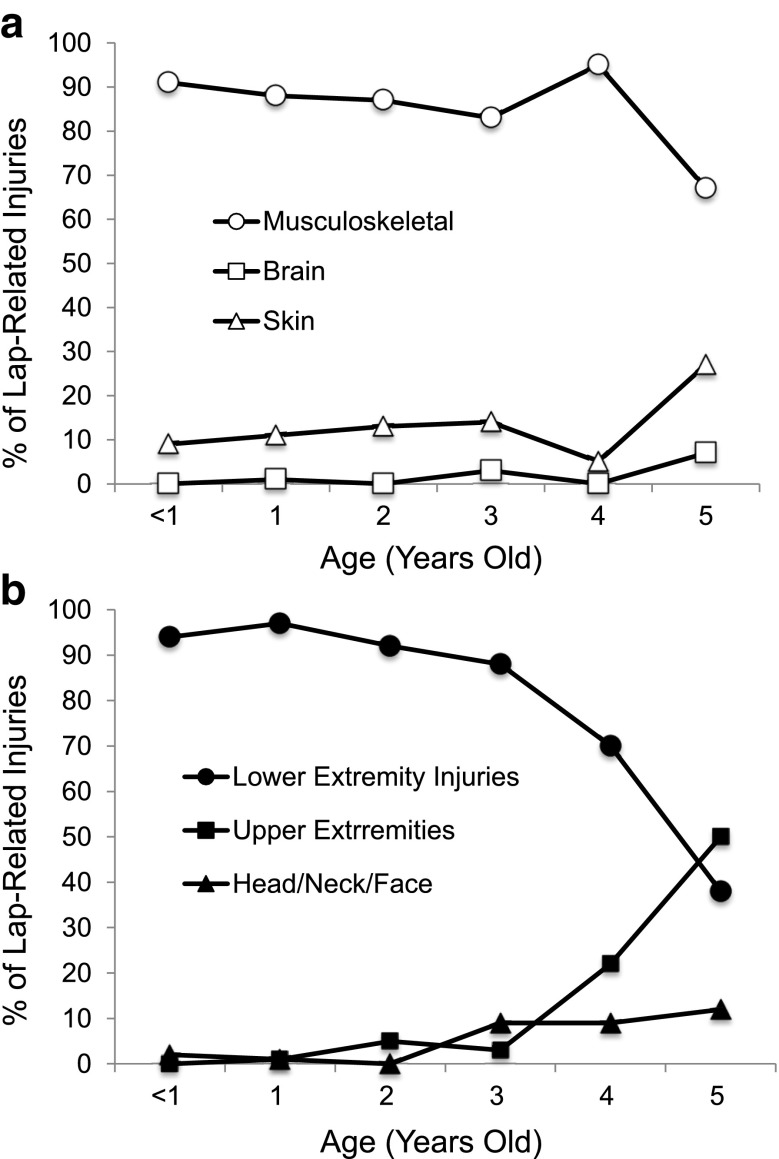
Percent of injuries in children identified as being on a lap at the time of injury. The percentage of children from < 1 to 5 years of age with the indicated lap-related injury diagnoses (Panel **a**) and the indicated body parts injured (Panel **b**) are shown

For all ages, two-thirds or more of injuries sustained while on a lap were musculoskeletal (Fig. 2a). However, nearly all of the musculoskeletal injuries were to the lower extremities in children < 4 years old, whereas an increasing proportion were to upper extremities in children 4–5 years of age (Fig. 2b).

Bivariate comparisons of cases identified in the NEISS narratives as occurring on a lap and those that were not showed a number of significant differences (Table 4). Lap-related injuries were almost twice as likely to involve children who were white than children of another race, nearly 5 times more likely to occur in a park/public area than in other locations, and approximately 7 times more likely to result in musculoskeletal injuries than in other injury types. The most striking comparison was that children identified as being on a lap at the time of their injury were over 40 times more likely to have a lower extremity injury than an injury to other body parts. In addition, those diagnosed with a fracture and noted to have been on a lap in the narratives were 50 times more likely to have a lower leg/ankle fracture than fractures involving other body parts.

**Table 4:** Comparative analysis of all pediatric injuries by whether patient was identified in the NEISS narrative as sitting on a lap. The indicated pairwise comparisons were performed using the chi square test

	Identified as Patient on Lap		Pearson χ^2^ Test		
	No^a^	Yes	OR	95% CI	*P* value
	*n* (column%)	*n* (column%)			
Sex					
Male	7069 (59%)	379 (59%)	1.01	0.86–1.19	0.92
Female	4970 (41%)	264 (41%)			
Race					
White	5656 (65%)	342 (77%)	1.81	1.44–2.26	< 0.0001
Other Race (ref)	3076 (35%)	103 (23%)			
Diagnosis					
Musculoskeletal	5522 (50%)	494 (88%)	7.12	5.53–9.16	< 0.0001
Other Injury Type (ref)	5574 (50%)	70 (12%)			
Brain	1271 (11%)	5 (1%)	0.069	0.029–0.17	< 0.0001
Other Injury Type (ref)	9825 (89%)	559 (99%)			
Skin	4294 (39%)	65 (12%)	0.21	0.16–0.27	< 0.0001
Other Injury Type (ref)	6802 (61%)	499 (88%)			
Body Part					
Lower Extremities	2734 (23%)	597 (93%)	43	32.0–58.0	< 0.0001
Other Body Parts (ref)	9263 (77%)	47 (7%)			
Upper Extremities	4142 (35%)	24 (4%)	0.073	0.049–0.11	< 0.0001
Other Body Parts (ref)	7844 (65%)	620 (96%)			
Head/Neck/Face	4629 (39%)	12 (2%)	0.030	0.017–0.054	< 0.0001
Other Body Parts (ref)	7357 (61%)	632 (98%)			
Torso	492 (4%)	11 (2%)	0.41	0.22–0.74	0.0024
Other Body Parts (ref)	11,494 (96%)	633 (98%)			
Fractures Only^b^					
Lower Leg and Ankle	1018 (24%)	336 (94%)	49.5	31.7–77.4	< 0.0001
Other	3152 (76%)	21 (6%)			
Disposition					
Stayed/Transferred	612 (5%)	10 (2%)	0.29	0.16–0.55	0.0012
Left ED (ref)	11,428 (95%)	634 (98%)			
Location					
Private Residence	1607 (19%)	29 (7%)	0.34	0.23–0.50	< 0.0001
Other Locations (ref)	6978 (81%)	372 (93%)			
School/Daycare	2090 (24%)	4 (1%)	0.031	0.012–0.084	< 0.0001
Other Locations (ref)	6495 (76%)	397 (99%)			
Park/Public Area	8585 (70%)	368 ((92%)	4.8	3.36–6.87	< 0.0001
Other Locations (ref)	3697 (30%)	33 (8%)			

*Abbreviations*: *ED* emergency department, *OR* unadjusted odds ratio, *CI* confidence interval

^a^Reference = Cases where the narrative did not directly state that the injury occurred while the patient was going down a slide on another person’s lap

^b^Children with fractures, *N* = 4527

### Regression analysis with age as an outcome

When children < 3 years old were compared to children 3–5 years of age using logistic regression analysis to identify independent determinants, the younger children were less likely to be male than female (Table 5). Whereas there was no difference by race in this comparison, if the model compared children < 2 years old to children 2–5 years of age, then the younger group was 1.26 times more likely to be white than to be another race as compared to the older group. In addition, children < 3 years old were more than twice as likely as 3-5 year olds to be injured at a private residence or at a park/public area than at a school or daycare center.

**Table 5:** Results from binary logistic regression analysis of playground slide-related injuries. Regression analysis was performed for the indicated outcomes. Outcome 1: injured child being < 3 years old as compared to 3–5 years of age. Outcome 2: Cases identified where the child was on a lap at the time of injury versus not identified as lap-related

	Likelihood of patient being < 36 months old^a^		Likelihood patient identified as sitting on a lap when injured^b^	
Variable	aOR	95% CI	aOR	95% CI
Sex				
Male	0.84	0.74–0.94	0.81	0.61–1.07
Female	1.0 (ref)		1.0 (ref)	
Age				
< 1 year	Not Applicable		11.5	4.6–28.9
1 year			5.43	2.57–11.5
2 years			2.62	1.19–5.78
3 years			1.84	0.77–4.38
4 years			0.89	0.31–2.50
5 years			1.0 (ref)	
Race^c^				
White	1.10	0.98-1.24	1.50	1.09–2.07
Other	1.0 (ref)		1.0 (ref)	
Location				
Private Residence	2.57	2.15–3.08	3.02	0.87–10.5
Park/Public Area	2.25	1.94–2.62	10.5	3.31–33.6
School/Daycare	1.0 (ref)		1.0 (ref)	
Diagnosis^d^				
Musculoskeletal	1.36	1.12-1.64	2.15	1.42–3.26
Brain	1.34	1.11–1.63	1.80	0.40–8.11
Skin	1.0 (ref)		1.0 (ref)	
Body Part				
Lower Extremities	7.71	5.04–11.8	4.10	1.24–13.5
Upper Extremities	1.37	0.89–2.10	0.29	0.08–1.10
Head/Neck/Face	2.62	1.74–3.96	0.20	0.044–0.95
Torso	1.0 (ref)		1.0 (ref)	
Disposition				
Stayed/Transferred	0.52	0.38–0.71	0.80	0.30–2.15
Left ED	1.0 (ref)		1.0 (ref)	
Identified as on lap				
Yes	3.99	2.69–5.93	Not Applicable	
No	1.0 (ref)			

^a^Outcome reference = Patients 3–5 years old; Number of cases in model = 5990; Cases not included in the model if they had missing data for one or more of the variables in the model

^b^Outcome Reference = Injury not identified as occurring when patient was on another person’s lap; Number of cases in model = 5990; Cases not included in the model if they had missing data for one or more of the variables in the model

^c^When the model compared children < 2 years old with children 2–5 years old, the younger age was more likely to be white: aOR, 1.26; 95% CI, 1.08–1.46

^d^For the model with age as outcome, only 9 cases were diagnosed with internal organ injury, a number too small to generate aOR and 95% CI

As compared to older preschoolers, children < 3 years of age were approximately 30% more likely to be diagnosed with a musculoskeletal or brain injury than with skin-related injuries. For body part injured, the younger children were over 7.5 times more likely than those 3-5 years of age to have a lower extremity injury and more than 2.5 times more likely to have an injury to the head, neck, or face than to have torso injuries. The younger age group was about half as likely to be hospitalized or transferred as the older age group. Finally, injuries in children < 3 years old were 4 times more likely than those in 3–5 year olds to be identified as lap-related.

### Regression analysis with being on a lap as an outcome

Lap-related injuries were not different from other cases by sex, but were 50% more likely to involve a white child than a child of another race (Table 5). Injuries sustained while a child was going down a slide on another person’s lap were 10 times more likely to occur in a park/public area than at other locations as compared to cases not identified as lap-related. Injuries occurring while on a lap were more than twice as likely to be musculoskeletal injuries as other injury types and more than four times more likely to result in lower extremity injuries than in injuries to other body parts. Injuries to the head/neck, and face were less likely to have occurred when the child was identified as being on a lap.

## Discussion

Using a national dataset of slide-related injuries, we found a number of significant differences between younger and older preschool children, including the injury mechanisms and outcomes. The most striking was the much higher likelihood of younger injured children having been on a lap when going down the slide and having suffered a lower extremity injury, in particular, a lower leg/ankle fracture. These findings are consistent with our stated hypothesis.

Previous studies on playground injuries have found the major mechanism of slide-related injury to be a fall (Vollman et al., 2009; Bae et al., 2017; Bernardo et al., 2001; Mayr et al., 1995; Petridou et al., 2002). Falls from the ladder, over the edge of the slide, or upon exiting the slide to the ground can all result in injuries to the head and face, or to the upper extremity from trying to absorb the forces of the fall and protect the head. In fact, these types of injuries were the most common ones for the older preschool children in our study with 42.5% and 40.7% of children 3–5 years of age having an upper extremity and head/neck/face injury, respectively. Other studies have found similar findings with US children of all ages (< 18 years) most frequently having slide-related upper extremity (40.4%) and head and neck (32.6%) injuries (Loder, 2008). Moreover, falls from slides have been shown to have a greater risk of severe injuries and fractures as compared to falls from other playground equipment (Keays and Skinner, 2012). Having more fall-related injuries may explain, at least in part, the higher proportion of older preschoolers in our study who were hospitalized or transferred as compared to their younger peers.

Although industrial and federal interventions by the American Society for Testing and Materials (ASTM) (American Society for Testing and Materials International, n.d.-a; American Society for Testing and Materials International, n.d.-b) and the U.S. CPSC (U.S. Consumer Product Safety Commission, 2010) have had a positive effect on playground design and surfacing, there have been increasing numbers of overall playground equipment-related injuries in the US since 2006 (Vollman et al., 2009; Cheng et al., 2016). Similarly, our investigation found that the number of slide-related injuries in children less than 6 years of age increased during the study period. This suggests greater efforts are needed to prevent these injuries, including further design and testing of playground surfaces.

Although the older preschool children in our study had slide-related injury patterns very similar to those seen in previous reports studying children of all ages, the younger children in our study were significantly different. In regression analysis, we found that children < 3 years of age were about 30% more likely to have a brain injury than to have skin-related diagnoses and 2.5 times more likely to have an injury to the head/neck/face region than to the torso as compared to preschoolers 3-5 years old. This is consistent with previous injury research that showed preschool children < 3 years of age to be more prone to head injuries than those ≥3 years old (Burrows et al., 2015; Joeris et al., 2014; Thomas et al., 2013). Similarly, in a study of Korean playground equipment-related injuries, children < 3 years old were nearly twice as likely to have a TBI as those 3–7 years of age (Bae et al., 2017).

In contrast, the markedly higher overall proportion of lower extremity injuries suffered by younger children as compared to older preschoolers has not previously been reported in a national sample. Additionally, we show that the percentage of children with slide-related injuries involving their lower extremity increased dramatically as age decreased from 15% for 3-year-olds to 82% for children < 1 year of age. A large proportion of these injuries were fractures involving the lower leg and ankle.

Utilizing keyword searches of the NEISS injury narratives, we were able to confirm 644 cases where the child had been on another person’s lap at the time of their injury. As expected, the percentage of children documented as being on a lap markedly increased as age decreased. Moreover, nearly all (93%) of those documented as being on a lap had lower extremity injuries. In fact, children < 3 years old and on a lap were > 40 times more likely to have an injury to the lower extremity than to other body parts and 50 times more likely to have a lower leg/ankle fracture than fractures of other bones as compared to older preschool children.

Although many of the children under 3 years of age were not documented as having been on a lap at the time of their injury, the overall frequency of lower extremity injuries and of lower leg/ankle fractures in these children was markedly similar to that of children documented as being on a lap. Thus, it seems reasonable to hypothesize that many of the injuries involving younger children that were not documented as being on a lap were in fact by a similar mechanism, i.e. lap-related.

What mechanism might explain this lap-related injury pattern? When a young child is going down a slide on the lap of another person, their foot may catch on the slide’s surfaces including the inner side or bottom of the slide. The lower leg can then twist and be pulled backward (sometimes becoming stuck under the adult) as both proceed down the slide. The much greater weight of an adult driving the downward momentum on the slide can create significant forces on the child’s lower extremity and result in a fracture, usually to the tibia. On the other hand, if a child goes down a slide by themselves, they are not likely to get a severe leg injury even if their foot catches due their relatively small size and weight.

A significant percentage of lower leg/ankle fractures were noted in children < 3 years of age, but this is likely to be an underestimate as many tibia fractures in young children are missed or not diagnosed on their initial visit to the ED. The type of lower extremity fracture found in patients with lap-related injuries in the case series reported by Gaffney were spiral fractures of the tibia (Gaffney, 2009), commonly called a toddler’s fracture. These fractures are often occult, meaning the initial x-ray fails to demonstrate the fracture line; only after some time can changes consistent with a fracture be appreciated on typical x-rays (Bauer and Lovejoy, 2017; Halsey et al., 2001; Shravat et al., 1996).

Many children with a lower extremity injury in the study who were < 3 years of age or on a lap were diagnosed as having a contusion, bruise, strain or sprain of the extremity. Unlike older children and adults, younger children are more likely to suffer a broken bone than a sprain or strain, as the most vulnerable part of their musculoskeletal system is their bones (Sharieff, 2017; Dinolfo, 2004). In addition, a simple bruise or contusion to a lower extremity is unlikely to cause a parent to bring a young child to the emergency room. The majority of these patients were probably not bearing weight, and it is likely that some, if not many, of these lower extremity injuries were actually occult fractures. In one study of toddler’s fractures, nearly 30% of them were initially diagnosed as a soft tissue injury and later showed radiographic evidence of a healing fracture (Shravat et al., 1996).

Most adults may be unaware of the risks associated with putting a young child on their lap to go down a slide. Few have received safety information or counseling regarding this issue, as many medical health care providers are equally unaware of the risk. Even injury prevention advocates have not recognized this as a risk factor when developing playground safety recommendations (Fuselli and Yanchar, 2012; KidsHealth, 2014; Safe Kids Worldwide, 2015).

Based on our findings, we would recommend that young children not go down a slide on another person’s lap as the safest, most effective approach to preventing these injuries. Families should be made aware of the dangers and counseled that if they elect to engage in this activity, extreme caution is necessary and that the child’s legs need to be secured such that their feet will not catch the slide’s surfaces.

### Limitations

Our study is limited to playground slide-related injuries that were seen in U.S. emergency departments participating in NEISS data collection, and thus may not be generalizable to other populations. Because a study variable to identify whether an injured child was on a lap at the time of the injury was not available, cases were identified from the NEISS crash narratives. Although younger children and those on laps had higher percentages of lower leg/ankle fractures, the NEISS database does not specify which bone was fractured. It is likely that most of the fractures designated as involving the lower leg and ankle were of the tibia and probably consistent with a toddler’s fracture, but the lack of database specificity in fracture diagnosis does not allow stating this explicitly. As narratives were not standardized, it is also highly likely that many cases of lap-related injuries were not identified. Other limitations of the NEISS database may include miscoding, as well as the potential for selection bias with regards to which caregivers decide to seek ED care for an injury. In addition, the database does not capture injuries evaluated outside the ED or those that receive no medical attention. Finally, the absence of exposure data to playground slides prevents any injury rate calculations.

## Conclusions

Decreasing age was associated with a higher likelihood of being identified as going down a slide on another person’s lap and a higher likelihood of lower extremity injuries, particularly fractures. A child’s foot can catch on the slide’s surfaces when going down on a person’s lap, and the subsequent twisting force may cause a broken bone of the lower leg. These fractures may in fact be underestimated in this patient population due to occult fractures of the tibia (toddler’s fractures), and healthcare providers should be mindful of the potential for these injuries. Parents should also be made aware of this increased risk, and advised that the safest approach is to not go down a playground slide with a young child on one’s lap. However, if a person elects to do so, they should be counseled to safely secure their child’s legs so that their feet cannot catch the slide’s surfaces.

## Abbreviations

aOR: Adjusted odd’s ratio
ASTM: American Society for Testing and Materials
CDC: Center for Diseases Control and Prevention
CI: Confidence interval
CPSC: Consumer Product Safety Commission
ED: Emergency department
NEISS: National Electronic Injury Surveillance System
OR: Unadjusted odd’s ratio
ref: Reference
SPSS: Statistics Package for the Social Sciences
TBI: Traumatic brain injury
U.S.: United States
